# Epitope of titin A-band-specific monoclonal antibody Tit1 5 H1.1 is highly conserved in several Fn3 domains of the titin molecule. Centriole staining in human, mouse and zebrafish cells

**DOI:** 10.1186/1747-1028-7-21

**Published:** 2012-09-17

**Authors:** Aavo-Valdur Mikelsaar, Alar Sünter, Ruth Mikelsaar, Peeter Toomik, Anu Kõiveer, Imre Mikelsaar, Erkki Juronen

**Affiliations:** 1 Institute of General and Molecular Pathology, University of Tartu, Tartu, Estonia; 2 LabAs Ltd, Tartu, Estonia; 3 Estonian University of Life Sciences, Tartu, Estonia

**Keywords:** Titin, Fn3 domains, Evolution, Human being, Mouse, Zebrafish (*Danio rerio*)

## Abstract

**Background:**

Previously we have reported on the development of a new mouse anti-titin monoclonal antibody, named MAb Titl 5 H1.1, using the synthetic peptide N-AVNKYGIGEPLESDSVVAK-C which corresponds to an amino acid sequence in the A-region of the titin molecule as immunogen. In the human skeletal muscles, MAb Titl 5 H1.1 reacts specifically with titin in the A-band of the sarcomere and in different non-muscle cell types with nucleus and cytoplasm, including centrioles. In this report we have studied the evolutionary aspects of the binding of MAb Tit1 5 H1.1 with its target antigen (titin).

**Results:**

We have specified the epitope area of MAb Tit1 5 H1.1 by subpeptide mapping to the hexapeptide N-AVNKYG-C. According to protein databases this amino acid sequence is located in the COOH-terminus of several different Fn3 domains of the A-region of titin molecule in many organisms, such as human being, mouse, rabbit, zebrafish (*Danio rerio*), and even in sea squirt (*Ciona intestinalis*). Our immunohisto- and cytochemical studies with MAb Tit1 5 H1.1 in human, mouse and zebrafish tissues and cell cultures showed a striated staining pattern in muscle cells and also staining of centrioles, cytoplasm and nuclei in non-muscle cells.

**Conclusions:**

The data confirm that titin can play, in addition to the known roles in striated muscle cells also an important role in non-muscle cells as a centriole associated protein. This phenomenon is highly conserved in the evolution and is related to Fn3 domains of the titin molecule. Using titin A-band-specific monoclonal antibody MAb Tit1 5 H1.1 it was possible to locate titin in the sarcomeres of skeletal muscle cells and in the centrioles, cytoplasm and nuclei of non-muscle cells in phylogenetically so distant organisms as *Homo sapiens*, *Mus musculus* and zebrafish (*Danio rerio*).

## Background

We have reported previously [1] on the development of a new mouse anti-titin monoclonal antibody, named MAb Titl 5 H1.1, using the synthetic peptide N-AVNKYGIGEPLESDSVVAK-C corresponding to an amino acid sequence in the A-band of the titin molecule as immunogen. In the human skeletal muscle, MAb Tit1 5 H1.1 reveals a clearly striated staining pattem, reacting with the A-band of the sarcomere. The antibody reacts with titin in cytoplasm, nucleus and centrioles in all of non-muscle cell types investigated so far. In the present study we have restricted (narrowed down) the epitope of MAb Tit1 5 H1.1 to the hexapeptide N-AVNKYG-C by subpeptide mapping and performed immunohisto-and cytochemically studies of the of MAb Tit1 5 H1.1 epitope expression in phenotypically distant organisms such are human being *Homo sapiens*, mouse *Mus musculus* and zebrafish (*Danio rerio*).

## Results and discussion

### Specifying the epitope of MAb Tit1 5 H1.1 by subpeptide mapping

**Figure 1:**
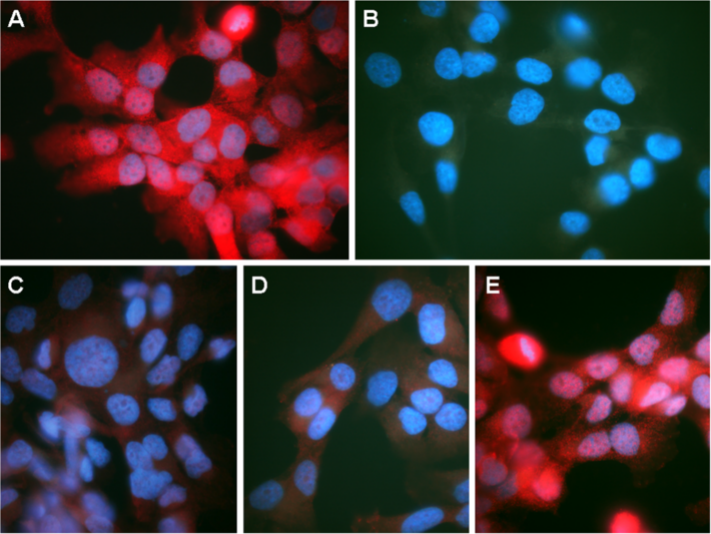
**Inhibition of the staining of the MAb Tit1 5 H1.1 by specific peptides. (A)** Location of MAb Tit1 5 H1.1 target antigen (titin) in Bowes melanoma cell line on the 2nd day of cultivation. The cells were fixed with 4% PFA and permeabilized with Tritone X-100 before staining for titin with MAb Tit1 5 H1.1. Specific staining was visualized with Alexa Fluor 594 conjugated to goat anti-mouse IgG (**red**), obj. 100x . **(B)** Negative staining of cells with secondary antibody. **(C)** Cells of the same culture as in **A** were stained with MAb Tit1 5 H1.1 but inhibited by specific full-sized peptide (N-AVNKYGIGEPLESDSVVAK-C). Note a marked reduction of staining intensity and no centriole staining,obj. 100x. **(D** and **E)** Cells were stained with MAb Tit1 5 H1.1 and inhibited by subpeptides N-AVNKYG-C and N-PLESDSVC, respectively. Note a marked reduction of staining intensity with inhibition using N-AVNKYG-C and no staining reduction with peptide N-PLESDSV-C, obj.100x. Cell nuclei were counterstained with DAPI (**blue**).

The full-size amino acid sequence originally used to prepare anti-titin monoclonal antibody MAb Tit1 5 H1.1 was a 19 amino-acid-long peptide with the sequence (NH2)AVNKYGIGEPLESDSVVAK(COOH) [1]. This amino acid sequence is located between positions 27969–27987 of the A- region of human full-size titin molecule corresponding for the most part to the C-terminus of fibronectin type-III domain 103(SMART:Fn3 domain annotation, SwissProt: Q8WZ42). Previously [1] we have shown that the full-size peptide incubated overnight with MAb 5 H1.1 inhibits fully its reaction with antigen. To narrow down the epitope of MAb Tit1 5 H1.1, the supernatants inhibited with subpeptides AVNKYG, IGEPLE, EPLESD, PLESDSV and ESDSVV were used in immunofluorescence studies. The N-AVNKYG-C was the only subpeptide that was able to inhibit the reaction of MAb 5 H1.1 with its antigen as did also the full-size peptide (Figure 1).

**Figure 2:**
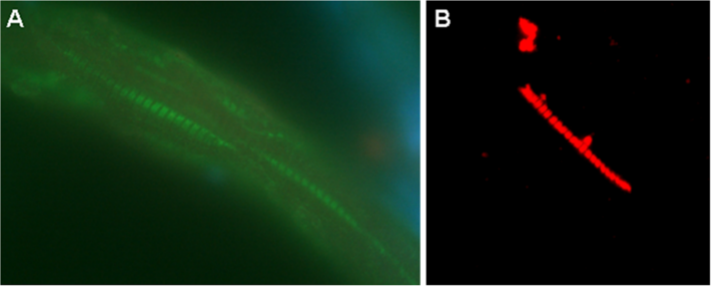
**Immunohistochemical staining of skeletal muscle biopsy from the back side of zebrafish with MAb Tit1 5 H1.1.** (**A**) Cryoslice of zebrafish skeletal muscle immunostained with MAb Tit1 5 H1.1, fixed with 4% PFA, specific staining (green) with Alexa 488, obj.100x. (**B**) Mechanically separated skeletal muscle fibre of zebrafish, fixed with 4% PFA, immunostained with MAb Tit1 5 H1.1, specific staining (red) with Alexa 594, obj.100x. Slices were embedded with Prolong Gold anti-fade reagent.

### Protein database analysis of the evolutionary relationship of the „narrowed”epitope AVNKYG of MAb Tit1 5 H1.1

To investigate the evolutionary relationship of the narrowed amino acid epitope (AVNKYG) of MAb Tit1 5 H1.1 between different organisms, the analysis of the information stored in protein databases was performed. The amino acid sequence N-AVNKYG-C was present in the C-terminus of several Fn3 domains of the human (Q8WZ42), mouse (A2ASS6), rabbit (Q28733-titin fragment), zebrafish (*Danio rerio*) (titin a) and even in sea squirt (*Ciona intestinalis*-UPI000180D3B0) titin molecule. (A blast sequence analysis of human proteins revealed the hexapeptide N-AVNKYG-C with 100% query coverage and 100% maximal identity only in 15 human titin isoforms, and 2 protein fragments of unknown origin which seem to be also fragments of titin molecule). We have used SMART:Fn3 domain annotation database for the numeration of Fn3 domains because according to that the first Fn3 domains from the N-terminus side of the titin molecule are fully comparable between the three species studied. In Protein Knowledgebase UniProtKB the number of Fn3 domains in the mouse (A2ASS6) is 134, and the numeration starts with No.1 in position 942–1037 and No. 2 in position 14343–14433. However, in SMART:FN3 domain annotation database these Fn3 domains do not exist at all, and our analysis has also shown that these Fn3 domains No. 1 and No.2 in the mouse do not have any amino acid homology either with human or zebrafish first Fn3 domains. According to SMART:FN3 domain annotation database the numeration of Fn3 domains can start from the No.1 in all of the three species, and the amino acid sequences of the corresponding Fn3 domains are fully comparable. However, in the zebrafish (*Danio rerio*) the Fn3 domain corresponding to Fn3 domain No.26 is fully absent, and so the numbers of the following Fn3 domains of the zebrafish are by one number lower compared to human and mouse titin. So, human and mouse Fn3 domain No.27 should be compared with the Fn3 domain number 26 in the zebrafish, and so on.

In the human titin molecule (Q8WZ42) we have found seven Fn3 domains with the amino acid sequence N-AVNKYG-C (Fn3 domains 19, 63, 71, 79, 87, 103 and 119). We have compared the amino acid sequences of these human Fn3 domains with the amino acid sequences of the corresponding Fn3 domains of the titin molecule in the mouse and the zebrafish (Table 1). In all these Fn3 domains, the mouse also has N-AVNKYG-C sequence, but in the four domains of zebrafish 1–2 amino acids are substituted by other amino acids. However, in the three remaining Fn3 domains all the three species carry N-AVNKYG-C sequence. The amino acid sequence homology for individual Fn3 domains (Table 2) between all three species was 51.1-79.8%, being the highest between human and mouse Fn3 domain number 87 and human and zebrafish number 86. The homology between human and mouse Fn3 domain No. 87 was 97,6%, and the homology beween mouse Fn3 domain No. 87 and zebrafish Fn3 domain No. 86 and between human Fn3 domain No. 87 and zebrafish Fn3 domain 86 was 86,9% and 84,5%, respectively. In general, this finding is in a good concordance with our knowledge of the evolutionary relationship between these three organisms. According to the present knowledge the last common ancestor of the zebrafish and human/mouse lived about 450 Ma ago and the mouse and human genomes diverged around 100 millions years ago [2-9]. An 83.1% frequency of conserved syntenies (physical co-localizations of genetic loci on the same chromosome) among the 804 orthologous gene pairs shared by humans and the zebrafish has been estimated, compared to 90.4% for 375 mouse-human gene pairs [5,6]**.** This is in quite a good accordance with our data on the homology of amino acid sequences between the corresponding Fn3 domains in the human, mouse and zebrafish titin molecule.

**Table 1:** Comparison of amino acid sequences between the corresponding Fn3 domains containing AVNKYG sequence in human (Q8WZ42),mouse (A2ASS6) and zebrafish (A5X6X5) titin molecule

**No’s of Fn3, species and location of Fn3 domains**		**Amino acid sequences of Fn3 domains of titin. The data according to SMART: Fn3 annotation**http:// http://smart.embl.de/smart/do_annotation.pl?DOMAIN=SM00060
19*	**H.s** 16425-16509	PS PPRN L AVTD IKAESC Y L T WDAPLD NGGSE I T H YVIDKR D ASRKKA EWE E VTNTAV E K RYG I W K L IPN G QYE FRV R **AVNKYG** IS
**19**	**M.m** 17287-17371	PS PPRN L AVTD IKAESC Y L TWDAPLD N GGSE I T H YIIDKR D ASRKKS EWEE VTNTAV E R RYG I W K L IPN G QYE FRV R **AVNKYG** IS
**19**	**D.r** 15268-15355	PT PPRN V AVSS IKAESCN L S WDAPLD I GGSEL T N YIVEMKD LNVEDP E KA E WVQVTKSII EK RYGVWN L VTG G NYKFRV K **A**E**NKYG**IS
**63**	**H.s** 22482-22565	PGPPEGPL A V TEV T S EKCVLSW F PPL D DGG A KI DH YI V Q K RETSRL A WTNVA SEV QV TKL KVTKLLKGNEYIFRV K **AVNKYG**VG
**63**	**M.m** 23344-23427	PGPPEGPL A V SDV T S EKCVLSW L PPL D DGG A KI DH YI V Q K RETSRL A WTNVA TEV QV TKL KVTKLLKGNEYIFRV M **AVNKYG**VG
**62**	**D.r** 21291-21374	PGPPEGPL H V TDM T V EKCVLSW L PPL H DGG G KI EY YI I Q R RETSRL T WTNVA TDL QV NRY KVTKLLKGNEYIFRV M **AVNKYG**VG
**71**	**H.s** 23546-23647	PGPP E GP VV I S GVT A EKC TLA WK P PL Q DGG SDIIN YIVERRETSRLVWT V V DAN VQTL SCKVT KLL E GNEY T FR IM **AVNKYG**V G
**71**	**M.m** 24426-24509	PGPP E GP VA I S GVT AEKC TLA WK P PL Q DGG SDITN YIVERRETSRLVWT L V DAN VQTL SCKVL KLL E GNEY I FR IM **AVNKYG**V G
**70**	**D.r** 22373-22456	PGPP D GP IS I Y GVT S EKC CIS WK T PL H DGG AEVSH YIVERRETSRLVWT V V ELK VQTL NLK IT KLL P GNEY I FR VI P**VNKYG** I G
**79**	**H.s** 24646-24729	PGPPEGP VQ V T GVT S EKC S L T W S PP LQ DGGS D IS H YV VE KRETSRLAWTVV ASEVVTNSL KVTKLL E GN E YV FR I M **AVNKYG** VG
**79**	**M.m** 25508-25591	PGPPEGP VQ V T GVT A EKC T L A W S PP LQ DGGS DIS H YV VE KRETSRLAWTVV ASEVVTNSL KVTKLL E GN K Y I FR I M **AVNKYG** VG
**78**	**D.r** 23454-23537	PGPPEGP LT V S GVT N EKC S L S W L PP RH DGGS S IS Y YV IQ KRETSRLAWTVV SGDCGATMFKVTKLL KGN E Y I FR VM **AVNKYG** VG
**87**	**H.s** 25729-25812	PGPP E GPLKVTGV T AEKCYL A W NP P LQ DGGA N ISHYIIEKRETSRLSWT Q V STEV QA LN YKVTKLLPGNEYIFRVM **AVNKYG**IG
**87**	**M.m** 26591-26674	PGPP E GPLKVTGV T AEKCYL A W NP P LQ DGGA S ISHYIIEKRETSRLSWT Q V SNEV QA LN YKVTKLLPGNEYIFRVM **AVNKYG**IG
**86**	**D.r** 24537-24620	PGPP D GPLKVTGV A AEKCYL H W SH P SH DGGA S ISHYIIEKRETSRLSWT V V EPKI QA IS YKVTKLLPGNEYIFRVM **AVNKYG**IG
**103**	**H.s** 27893-27976	PGPP GGP I E FK V TA EKI T LL W R PPAD D GGA KI THYIVEKRETSR VV WS MVS E HLEE CI IT TTKI IKGNEY I FRVR **AVNKYG**I G
**103**	**M.m** 28755-28838	PGPP GGP I E FK V TA EKI T L LW R PPAD D GGA KI THYIVEKRETSR VV WS MVA E NLEE CI T TTKI IKGNEY V FRVR **AVNKYG**I G
**102**	**D.r** 26701-26784	PGPP AGE I Q FK I TA DTM T IM W D PPAD E GGA MV THYIVEKRETSR IM WS IIS E KLQD CI T VPRLIKGNEY I FRVR G**VNK**H**G**VG
**119**	**H.s** 30068-30150	PGP CGKLTVS RVT Q EKCT LA W SL P Q EDGG AEIT HYIVERRETSRLNWVI V E G EC P T L S Y V VTR LIKNNEY I FRVR **AVNKYG**PG
**119**	**M.m** 30930-31012	PGP CGKLTV RVT E EKCT LA W S LP Q EDGG AEIT HYIVERRETSRLNWVI V E G EC L T A S Y V VTR LIKNNEY T FRVR **AVNKYG**LG
**118**	**D.r** 28873-28957	PGP PAGTITIS RVT D EKCT VS W K I P L EDGG DHVS HYIVERRETSRLNWVI M E T EC K T L S C V STK LIKNNEY I FRVR G**VNKYG**PG

*H.s* – *Homo sapiens*; *M.m* – Mus musculus; *D.r* – *Danio rerio* (zebrafish) * - the numbers of human and mouse Fn3 domains are coincidental; number 19 is the same in all species compared, but in zebrafish (*Danio rerio*) starting from Fn3 domain No.63 the numbers of corresponding Fn3 domains are smaller by one number (that is to human and mouse Fn3 domain No.63 corresponds to No.62 in zebrafish, and so on) . See also the explanation on the numeration of Fn3 domains in text. Homologous amino acids in corresponding Fn3 domains between all the three species are in boxes. AVNKYG sequences are underlined and in bold.

**Table 2:** Amino acid sequence homology (in%) between the corresponding Fn3 domains containing AVNKYG sequence in human (Q8WZ42),mouse (A2ASS6) and zebrafish (A5X6X5) titin molecule

**Species compared**	**The homology beween AVNKYG containing Fn3 domains in human (Q8WZ42), mouse (A2ASS6) and zebrafish (*Danio rerio*- A5X6X5) titin molecule**						
	19*	**63**	**71**	**79**	**87**	**103**	**119**
**H.s/M.m**	**96.5 (82/85)	**94.0** (79/84)	**94.0** (79/84)	**94.0** (79/84)	**97.6** (82/84)	**96.4** (81/84)	**94.0** (79/83)
**H.s./D.r.**	**46.6-48.2** (41/85-88)	**77.4** (65/84)	**67.9** (57/84)	**71.4** (60/84)	**82.1** (69/84)	**67.9** (57/84)	**72.6-73.1** (61/83-84)
**M.m/D.r.**	**47.7-49.4** (42/85-88)	**82.1** (69/84)	**67.9** (57/84)	**70.2** (59/84)	**83.3** (70/84)	**65.5** (55/84)	**70.2-71.1** (59/83-84)
**H.s/M.m/D.r**	**51.1**	**76.2**	**66.7**	**66.7**	**79.8**	**66.7**	**69.0**

*H.s* – *Homo sapiens*; *M.m* – *Mus musculus*;*D.r* – *Danio rerio* (zebrafish).* the numbers of human and mouse Fn3 domains compared are the same; number 19 is the same in all species compared, but in zebrafish starting from the Fn3 domain No.63 the numbers of corresponding Fn3 domains are by one number smaller. See also Table 1 and the explanation on the numeration of Fn3 domains in text. ** the percentage of homology is obtained by dividing the number of consensus amino acids by the whole number of amino acids in the Fn3 domain.

### Immunohistochemistry of human and zebrafish skeletal muscle biopsies using MAb Tit1 5 H1.1

Previously [1] we have shown that in the human skeletal muscle, MAb Tit15H1.1 reveals a clearly striated staining pattern reacting with the A-band of the sarcomere. Western blot and amino acid sequence analyses with the ESI-MS/MS of human skeletal muscle tissue samples proved the target antigen of MAb Tit15H1.1 to be titin. In this study we showed that in the skeletal muscle of zebrafish MAbTit15H1.1 also reveals the striated staining pattern like in the human skeletal muscle (Figure 2). This finding proves the presence of the epitope of MAb Tit1 5 H1.1 in zebrafish muscle tissue. Our colleagues have preliminary data on the existence of a regular granular staining pattern of the A-band of zebrafish skeletal muscles obtained by immuno-EM with *Nanogold labelled Fab-fragments of MAb Tit1 5 H1.1*, proving the accessibility of multiple epitopes in Fn domains to MAb Tit1 5 H1.1 (Masso R. et al., personal communication).

**Figure 3:**
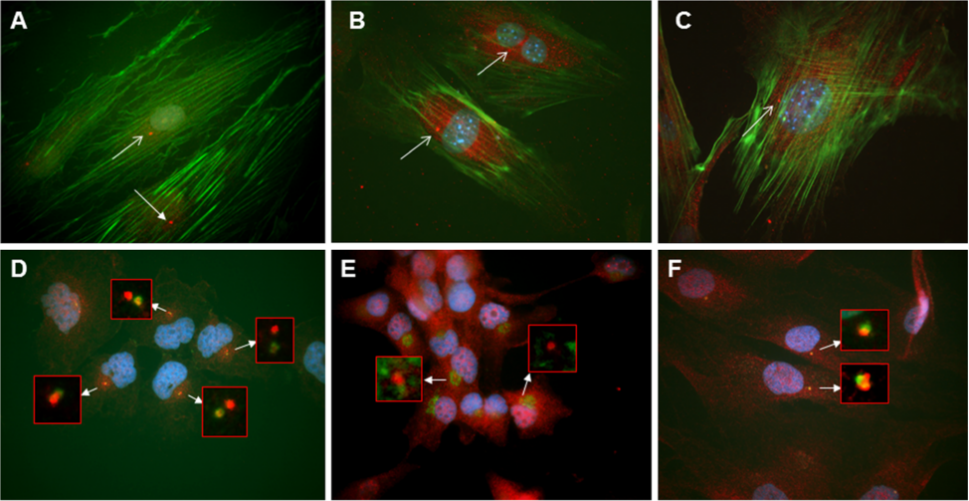
**Immunofluorescence co-localization of the MAb Tit1 5 H1.1 target antigen (titin) with respect to actin filaments and some centrosome/centriole proteins.** (A) Fibroblasts of the human adult fibroblast cell-line SA-54 were cultured for 3 weeks, fixed with 4% PFA,permeabilized, and double labeled with Alexa Fluor 488 conjugated to Phalloidin for F-actin (green) and with the MAb Tit1 5 H1.1 primary antibody for titin (specific staining detected with Alexa Fluor 594 conjugated to goat anti-mouse IgG secondary antibody (red). (B,C) Mouse embryonal fibroblasts (MEF7) cultured for 3 days, fixed with 4% PFA, permeabilized, and double labeled with Alexa Fluor 488 conjugated to Phalloidin for F-actin (green) and with the MAb Tit1 5 H1.1 primary antibody for titin (specific staining with Alexa Fluor 594, red). (D) Bowes melanoma cells cultured for 2 days, fixed with 4% PFA, permeabilized, and double labeled with MAb Tit1 5 H1.1 for titin (red) and with goat anti-human ninein for ninein (green), and (E) with MAb Tit1 5 H1.1 for titin (red) and with rabbit anti-human Î³-tubulin for Î³-tubulin (green). (F) Fibroblasts of the human adult fibroblast cell-line SA-54 were cultured for 3 weeks, fixed with 4% PFA, permeabilized, and double labeled with MAb Tit1 5 H1.1 for titin (red) and with rabbit anti-human pericentrin for pericentrin (green). Note the individual staining of the target antigens of all antibodies with centrioles/centrosomes. Arrows show centrosome/centriole staining. Cell nuclei were stained blue with DAPI (obj. 100x).

### Immunocytochemistry of cultured human, mouse and zebrafish (*Danio rerio*) cells

**Figure 4:**
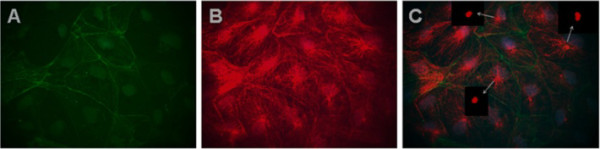
**Immunofluorescence co-localization of MAb Tit1 5 H1.1 target antigen (titin) with F-actin in different cells of zebrafish primary culture of the testes.** Cells were fixed on 3rd day of cultivation with 4% PFA, permeabilized, and double-labelled (**C**) with Mab Tit1 5 H1.1 for titin (red – Alexa 594) and with Phalloidin for F-actin (green – Alexa 488). Cell nuclei were stained blue with DAPI (obj. 100x). Arrows show centriole staining.In Photoshop image processing either red colour (**A**) or green colour (**B**) was removed and one can see independent staining both of F-actin (**A**) and titin (**B**). Note a very extensive fibrous centriole-orientated staining of titin.

**Figure 5:**
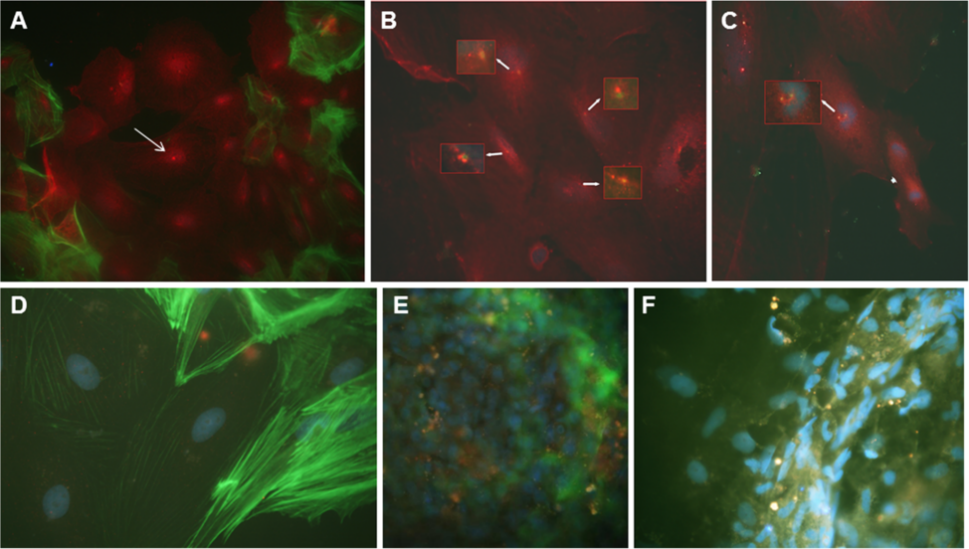
**Co-location of MAb Tit1 5 H1.1 target antigen (titin) with F-actin and Î³-tubulin, negative staining with secondary antibodies,and the inhibition of specific staining of MAb Tit1 5 H1.1 with subpeptide N-AVNKYG-C in cells of zebrafish.** (**A**) The cells of zebrafish testes were cultivated for 3 days, fixed, permeabilized, and double labeled with Alexa Fluor 488 conjugated to Phalloidin for F-actin (green) and with the MAb Tit1 5 H1.1 primary antibody for titin (specific staining with Alexa Fluor 594 conjugated to goat anti-mouse IgG secondary antibody (red). Arrow shows centriole staining. Note also a strong staining of nuclei and cytoplasm (obj. 40x). (**B**) The cells of zebrafish testes were double labeled with MAb Tit1 5 H1.1 for titin (red) and with rabbit anti-human Î³-tubulin for Î³-tubulin (green). Note the individual staining of the target antigens of both antibodies with centrioles/centrosomes. Arrows show centrosome/centriole staining.(**C**) Double labeling of mitotic cells of zebrafish with MAb Tit1 5 H1.1 for titin (red) and with rabbit anti-human Î³-tubulin for Î³-tubulin (green). The long arrow shows the co-location of MAb Tit1 5 H1.1 antigen (titin) and Î³-tubulin in the mitotic spindle with separated double-labeled centrioles (yellow stain), and the short arrow shows a late anaphase (only titin is labelled, red stain).(**D**) Negative staining of cells with secondary antibodies. (**E**,**F**) The cells were stained with MAb Tit1 5 H1.1 for titin but inhibited by the subpeptide N-AVNKYG-C. Note a full absence of staining with Alexa 594. Cell nuclei were stained blue with DAPI (obj. 100x).

Previuosly, we have shown [1] that MAb 5 H1.1 reacts with titin also in human non-muscle cells, producing a punctate pattern in cytoplasm and in the nucleus. The most striking finding was a clear reaction of MAb Tit1 5 H1.1 with centrioles in all cell types investigated. An immuno-cytochemical co-localization study with ninein-specific antibodies confirmed that the target antigen of MAb Tit1 5 H1.1 is a centriole-associated protein (ninein is a protein that in man is encoded by the *NIN* gene and is important for centrosomal function). The inhibition of titin synthesis using titin siRNA duplex for the destruction of titin mRNA showed a decreased staining of centrioles by MAb Titl 5 H1.1 in non-musde cells and thus supports the proposal that the target antigen of MAb is indeed titin. In the present study we investigated the association of titin with centrioles besides the human being also in mouse and zebrafish cultivated cells. In the human species we have studied the co-location of titin with F-actin in the cells of an original human adult fibroblast cell-line SA-54 cultured for 3 weeks and in the mouse embryonic fibroblasts of MEF7 cell-line cultured for 3 days. The staining pattern was similar between mouse and human cells: punctuated staining of titin in cytoplasm, a weak staining of nuclei and a very bright staining of centrioles. Only very thin actin threads seem to be co-stained with titin (Figure 3A-C). We have made in human cells the fluorescence co-localization study of MAb Tit1 5 H1.1 target antigen (titin) with such typical centrosome/centriole proteins as ninein, γ-tubulin and pericentrin (Figure 3D-F). In all cases the antigens showed individual staining patterns co-located with centrioles/centrosomes. In order to study the staining of titin with MAb Tit1 5 H1.1 in non-muscle cells of the zebrafish we have prepared short-time primary cultures of zebrafish’s inner organs, mainly from the testes. The cells felt themselves well in these conditions showing even several mitotic figures (Figure 5C). In Figure 4 zebrafish cultured cells double-labeled with Mab Tit1 5 H1.1 for titin and for F-actin a very bright staining of centrioles and an extensive centrosome-area-oriented fibrous staining of cytoplasm have been shown. In Figure 5 the co-location of MAb Tit1 5 H1.1 target antigen (titin) with F-actin (Figure 5A) and γ-tubulin (Figure 5B,C), a negative staining with secondary antibodies (Figure 5D), and the inhibition of specific staining of MAb Tit1 5 H1.1 with subpeptide N-AVNKYG-C in cells of zebrafish.(Figure 5E,F) are shown. As in human cells neither the secondary antibodies (negative control) nor MAb Tit1 5 H1.1 inhibited with the subpetide N-AVNKYG-C stained the cells. The double labelling with MAb Tit1 5 H1.1 for titin and with rabbit anti-human γ-tubulin for γ-tubulin revealed the individual staining of target antigens of both antibodies with centrioles/centrosomes in the interphase and mitotic cells.

**Figure 6:**
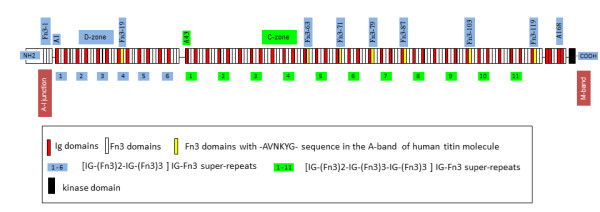
**IG-Fn3 domain organisation of the A-band in human titin molecule (Q8WZ42).** IG-like domains are shown as red boxes and Fn3-like domains in white. The titin kinase domain is colored black, and the Fn3-like domains containing N-AVNKYG-C sequence are shown in yellow. 1–6 in blue boxes are 7 domain super-repeats and 1–11 in green boxes are 11 domain super-repeats (modified according to [12]).

The main aim of this study was not a comparative interspecies analysis of the amino acid sequences of the titin whole molecule as such. Our aim was a search for the epitope sequence N-AVNKYG-C in the titin molecule of other organisms, and if found, to apply immunohisto-and cytochemical methods to explain whether or not the staining using MAb Tit1 5 H1.1 reveals a similar striated staining pattern in skeletal muscles and centriole staining as we had found earlier in the human being. The amino acid sequence N-AVNKYG-C can be found in several Fn3 domains in the titin molecule of different organisms. Immunohistochemical staining of the skeletal muscle biopsy from the back side of the zebrafish with MAb Tit1 5 H1.1 (Figure 2) gave a similar striated sarcomere staining pattern as in the human being. Costa and co-workers [10] revealed a striated pattern of myoblasts during myofibrillogenesis in the zebrafish stained for titin using anti-titin monoclonal antibody clone T11 but no centriole staining was found. Further, we have demonstrated that centrioles also can be stained in mouse embryonic fibroblast cells (Figure 3) and most interestingly also in short-term cultures of testicular cells of the zebrafish (Figures 4 and 5). In these cells there was also a noteworthy staining of nuclei and cytoplasm with a strong centriole-orientated fibrous staining. In human cells we had not detected such a fibrous staining of cytoplasm therefore this phenomenon needs further investigation. Both in human (Figure 1) and zebrafish cells (Figure 5C-D) a specific staining of MAb Tit1 5 H1.1 was fully inhibited (a full reduction of staining intensity and no centriole staining) by the incubation with the subpeptide N-AVNKYG-C. The accessibility of MAb Tit1 5 H1.1 to the hexapeptide epitope could be explained by its surface location. Muhle-Goll and co-workers [11] have determined, using NMR, the structure of the titin Fn3 module A71 and shown that many of its conserved residues (including N-AVNKYG-C-sequence – A-V.M.) are exposed on the surface of the domain, grouped together at one side [12]. IG and Fn3 domains form connections called super-repeats in the A-region. In the D-zone there are super-repeats in which seven individual IG-Fn3 repeats are arranged in a [IG-(Fn3)2-IG-(Fn3)3]n pattern. The C-zone of the A-region contains eleven individual repeats in a [IG-(Fn3)2-IG-(Fn3)3-IG-(Fn3)3]n pattern [12-18]. Interestingly, in all cases where the N-AVNKYG-C sequence was present, it was located in the C-terminal part of the first Fn3 domain following the first IG-domain of the 7- domain super-repeats in the D-zone (Fn3 domain No.19) and in C-zone following the first IG-domain of the 11- domain super-repeats (Fn3 domains 63, 71, 79,87, 103 and 119) (Figure 6). We speculate that this specific location of the repeated N-AVNKYG-C sequence may be in some way connected with the spatial configuration of super-repeats in the association of titin with centrioles. The antigen (titin) stained by MAb Tit1 5 H1.1 is always tightly connected with centrioles. We can propose that the A-region of titin covering the centriole brings the kinase domain located near the C-terminus of A-region (Figure 6) into the contact with centrioles that may be important for their function. Earlier the association of titin with myosin has been shown [12,19-21]. Muhle-Goll and co-workers [12] have shown a direct association of myosin with four Fn3 complex fragments A77-78, A80-82 and A84-86 which are adjacent to Fn3 domains 79 and 87 containing N-AVNKYG-C sequence. And as it was also shown earlier [22] that myosin is associated with mammalian centrosomes, we could propose that titin/myosin interaction may also play some important role in the function of centrioles. However, it needs a detailed further investigation because many other proteins are also involved in the formation of centrosomes. One can wonder why other anti-titin antibodies have not shown the co-location of titin with centrioles. We have tried to co-locate titin with centrioles with the some other commercial anti-titin antibodies (anti-PEVK MAb 9D10, Developmental Studies Hybridoma Bank, University of Iowa), MAb Tit2 2E8.1 against PEVK region of titin (our original antibody), anti-titin MAb 1553 (clone 9B9, Millipore), anti-titin MAb T11 (ab7034, Abcam), anti-titin rabbit polyclonal antibody H-300 (epitope corresponding to amino acids 33124–33423 mapping at the C-terminus of titin of human origin, Santa Cruz Biotechnology, Inc.). Any of these antibodies did not show co-location (co-staining) with centrioles (unpublished data). It may be that in non-muscle cells the titin exists mainly as different small isoforms with different epitopes which can not be caught by the antibodies tested. In favour of this proposal are also the results of our Western blot experiments with MAb Tit15H1.1 revealing in non-muscle cells (including, fibroblasts, testis, etc.) instead of the band of full-size titin several smaller immunopositive bands which may be interpreted as smaller isoforms of titin (unpublished data). We have still not found any information in the literature proving that titin is a regular centriolar/centrosome protein, not even in the centrosome proteomics database [23]. However, in one work it was mentioned that Ce-titin in *C.elegance* can take part in centrosome formation.[24]. The authors showed that the Ce-titin epitope EU102 moved during mitosis from the nuclear membrane to the mitotic spindle and/or centrosomes and was then either transiently dispersed or masked.

## Conclusions

In conclusion, we have succeeded in narrowing down the peptide epitope of our titin A-band-specific monoclonal antibody Tit1 5 H1.1 from 19-aa-peptide N-AVNKYGIGEPLESDSVVAK-C to hexapeptide N-AVNKYG-C. This peptide sequence proved to be highly conserved in several corresponding Fn3 domains of titin in different organisms. It was possible by using MAb Tit1 5 H1.1 to locate titin immunohisto-and cytochemically both in the sarcomeres of skeletal muscle cells and in centrioles, cytoplasm and the nuclei of non-muscle cells in phylogenetically so distant organisms as are *Homo sapiens*, *Mus musculus* and zebrafish (*Danio rerio*). These findings prove that titin has been a very ancient component of the centrosome.

## Materials and methods

### Human cell cultures

In this study an original normal human adult skin cell culture SA-54 (developed by LabAs Ltd.) and the commercial cell-line human melanoma Bowes were used. The cell lines were grown in a medium containing DMEM/F12 supplemented with 10% of FCS and gentamycin (all Invitrogen, GIBCO).

### Mouse cell cultures

Mouse embryonic fibroblast culture MEF7 was used and prepared according to the prescription of Boris Greber (Isolation and handling of primary mouse embryonic fibroblasts (MEFs) *accompanying protocol to “Mouse embryonic stem (ES) cell culture - basic procedures”*http://www.molgen.mpg.de/~rodent/MEF_protocol.pdf).

### Zebrafish skeletal muscle biopsies, and short-term cell cultures of non-muscle cells

#### **Zebrafish (*Danio rerio*)**

Adult Zebrafishes (*Danio rerio*) were purchased from a licensed zooshop.

The research was approved under animal care permit No.102 by the Commission of the Authorization of Animal Testing Permits of the Estonian Ministry of Agriculture.

#### ***Zebrafish organs for immuno-and cytochemistry***

Fishes were fasted for at least 24 h, anesthetized in 0.2% Tricaine (ethyl 3-aminobenzoate methanesulfonate salt, Tricaine MS-222, Fluka, cat.no. A5040) and then euthanized by incubation in ice water for 15 min. Tricaine was added directly to the anaesthetic bath. An euthanized male fish was rinsed once with 70% ethanol and placed into a sterile PBS solution with antibioticum gentamycin in a Petri dish. The skin and muscle of the fish were carefully removed from the ventral wall and internal organs visualized as described by Gupta and Mullins [25]. Spinal muscle tissue biopsies were taken for immunohistochemistry and testes were removed for a short-term cell culture.

#### ***Short-term cultures of zebrafish testes***

The testes were removed and placed into a sterile Petri dish with sterile PBS supplemented with gentamycin. Thereafter testes were minced and trypsinized for 5 min with a mixture of 0.05% trypsin with 0.53 mM EDTA. The cell suspension (suspension contained also some small cell clusters) was washed for 3 times by centrifugation at 100 *g* for 5 min with DMEM:F12 medium containing 10% of FCS and gentamycin (Gibco, Invitrogen) and seeded to grow on the cover-glasses in 6-well plates (Nunc) in DMEM:F12 medium containing 20% of FCS and gentamycin (all Invitrogen, GIBCO). The cells were cultured in CO_2_ incubators at 37°C for 1–3 days.

#### ***Immunohistochemistry of zebrafish skeletal muscle***

Zebrafish skeletal muscle cryoslices were prepared from skeletal muscle biopsies using the embedding medium (Thermo Shandon, Pittsburgh,PA). Sections were cut by using cryostat Cryocute E. Reichert-Jung at a thickness of between 8 and 10 μm. The sections were fixed with 4% PFA in PBS for 30 min at 4°C, washed for 3×5 min with PBS and incubated with the hybridoma supernatant of anti-titin MAb Titl 5 H1.1 for 1 h at RT, then washed for 3×5 min with PBS. The reaction was visualized by using Alexa 594 or A488 fluorochrome-conjugated goat secondary anti-mouse IgG antibody (Invitrogen, Molecular Probes, Eugene, Oregon, USA). The cell nuclei were counterstained with DAPI. The preparations were mounted in the anti-fading mounting medium Prolong Gold Antifade (Molecular Probes) and covered with coverslips. The immunoreaction was checked by a visual microscoping system (Olympus BX, using objectives UplanFI 20x/0.50, 40x/0.75, or 100x/1.30 *Oil* Iris, and the Olympus DP50-CU Photographing System, Tokyo, Japan).

#### ***Immunocytochemistry of cells grown in vitro***

Paraformaldehyde (PFA) fixation method was used. In PFA fixation the coverslips with the growing cells were transferred without any previous washing into dishes containing pre-warmed 4% paraformaldehyde in PBS and left for 5 min at room temperature. Then the coverslips were washed three times for 5 min each with PBS, and the excess of aldehyde was quenched with 50 mM NH_4_Cl in PBS (10 min). After washing twice with PBS, the cells were permeabilized for 10 min with 0.1% Tritone X-100 in PBS, washed with PBS and blocked. The coverslips were then transferred into a blocking solution (0.3% casein, 0.01% Tween-20 in PBS) for 1 h at room temperature or overnight at 4°C. The blocking solution was removed by aspiration, and the cells were stained as follows. The cells were incubated for 1 h at RT with the MAb Tit1 5 H1.1 supernatant. The coverslips were washed at least three times for 5 min each with PBS, and immunolabeling was visualized by incubating the cells with the secondary goat anti-mouse antibody conjugated with fluorochrome Alexa 594 (cat.no. A11032, Molecular Probes) for 1 h at RT. In all cases, the coverslips were washed at least three times for 5 min with PBS, 10 μL of DAPI solution (1 mg/mL) was added into the last PBS, and then the coverslips were incubated for 5 min at RT. After quick rinsing in distilled water, the coverslips were mounted in the anti-fading mounting medium Prolong Gold Antifade (Molecular Probes). The cells were checked by a visual microscoping system (Olympus BX, using objectives UplanFI 20x/0.50, 40x/0.75, or 100x/1.30 Oil Iris, and Olympus DP50-CU Photographing System). Phalloidin conjugated to Alexa 488 (Molecular probes) was used as marker for F-actin (Molecular Probes). The following primary antibodies were used for the immunofluorescence co-localization of the target antigen (titin) of MAb Tit1 5 H1.1 with centrioles/centrosomes: goat polyclonal antibody to human ninein and rabbit polyclonal antibody to human γ-tubulin (both from Santa Cruz Biotechnology,Inc., cat.no. sc-50142 and sc-10732, respectively), and rabbit polyclonal antibody to human pericentrin (cat.no. ab4448, AbCam, Cambridge, UK). The specific staining of ninein was visualized with Alexa 488 conjugated to donkey anti-goat secondary antibody (cat.no. A11055, Molecular Probes) and the specific staining of γ-tubulin and pericentrin was visualized with Alexa 488 conjugated to goat anti-rabbit secondary antibody (cat.no. A11008, Molecular Probes). We have used for the co-localization study of titin and γ-tubulin the same rabbit anti-human γ-tubulin polyclonal antibody (sc-10732, H-183, Santa Cruz Biotechnlogy, Inc.) that is developed against a synthetic peptide corresponding to last 183 amino acids in C-terminal part of human gamma-tubulin (tubulin gamma-1 chain, human, P23258, UniProtKB/Swiss-Prot) and used by us for human cells. This amino acid sequence has 96,2% of homology with zebrafish gamma-tubulin (tubulin gamma-like,*Danio rerio*, Q7ZVM5, UniProtKB/TrEMBL), and the anti-human gamma-tubulin antibody works well also in zebrafish cells.

#### ***Epitope analysis of MAb Tit1 5 H1.1 by subpeptide mapping***

The amino acid sequence originally used to prepare anti-titin monoclonal antibody MAb Tit1 5 H1.1 was a 19-amino-acid-long peptide with the sequence (NH2)AVNKYGIGEPLESDSVVAK(COOH), which is for the most part located in the C-terminus of fibronectin type-III domain 103 of the A-band of a full titin molecule (SwissProt: Q8WZ42). To narrow down the epitope of MAb Tit1 5 H1.1 the following subpeptides were used: AVNKYG, IGEPLE, EPLESD, PLESDSV and ESDSVV. The peptides were synthesized by Inbio Ltd. (Tallinn, Estonia). Inhibition of MAb Tit1 5 H1.1 by subpeptides was performed incubating 1,0 mL of the supernatant of MAb Titl 5 H1.1 overnight with 2 mg of each peptide. This was considered to give ~ 100 x overweight of the peptide compared to the amount of MAb calculated to be 20 μg per 1 mL as a maximum. The supernatants with inhibited MAb Titl 5 H1.1 were used in immunofluorescence studies.

#### ***Protein database analysis of the evolutionary relationship of the “narrowed”epitope N-AVNKYG- C of MAb Tit1 5 H1.1***

The following protein databases were used to investigate the evolutionary relationship of the narrowed amino acid epitope (AVNKYG) of MAb Tit1 5 H1.1 with other organisms: PROWL of the Rockefeller university http://prowl.rockefeller.edu/prowl/proteininfo.html, Protein Knowledgebase UniProtKB http://www.uniprot.org/, SMART:FN3 domain annotation http://smart.embl.de/smart/do_annotation.pl?DOMAIN=SM00060 and Wellcome Trust Sanger Institute http://pfam.sanger.ac.uk/.

## Competing interests

The authors declare no competing interests.

## Authors’ contributions

A-VM -designed the study, performed immunocytochemical analysis, performed protein database analysis and prepared the manuscript AS - participated in protein database analysis and drafting the manuscript RM - participated in cell culture study, involved in drafting the manuscript PT - participated in the protein database analysis and drafted the manuscript AK - participated in immunohistochemical studies IM - participated in zebrafish cell culture studies and analysis EJ - participated in study design and drafted the manuscript. All authors read and approved the final manuscript.

## Authors’ information

**A.V.M**. is professor in human biology and genetics at the Institute of General and Molecular Pathology of Medical faculty of the University of Tartu, his main scientific interests are connected with human genetics, medical genetics, cell biology and immunobiotechnology.

**AS** (Mag of Sci) is a researcher in human biology and genetics at the Institute of General and Molecular Pathology of Medical faculty of the University of Tartu. His main scientific interests are connected with proteomics and cell biology.

**RM (**MD, PhD**)** is Ass. Professor in medical genetics at the Institute of General and Molecular Pathology of Medical faculty of the University of Tartu. Her main scientific interests are connected with cytogenetics, medical genetics and cell biology.

**PT** (Mag Sci) is a researcher at Estonian Life Science University, specialist in proteomics and using of ESI-MS/MS.

**AK** is a specialist in immunohistochemistry at the Institute of General and Molecular Pathology of Medical faculty of the University of Tartu

**IM** is a laboratory specialist in the field of ichthyology at LabAs Ltd

**EJ (**PhD**)** is a senior researcher**,** at the Institute of General and Molecular Pathology of Medical faculty of the University of Tartu. His main scientific interest are connected with cell biology, immunobiotechnology
